# Exploring Novel Frontiers: Leveraging STAT3 Signaling for Advanced Cancer Therapeutics

**DOI:** 10.3390/cancers16030492

**Published:** 2024-01-24

**Authors:** Taiwo Adesoye, Debasish Tripathy, Kelly K. Hunt, Khandan Keyomarsi

**Affiliations:** 1 Department of Breast Surgical Oncology, The University of Texas MD Anderson Cancer Center, Houston, TX 77030, USA; khunt@mdanderson.org; 2 Department of Breast Medical Oncology, Division of Cancer Medicine, The University of Texas MD Anderson Cancer Center, Houston, TX 77030, USA; dtripathy@mdanderson.org; 3 Department of Experimental Radiation Oncology, The University of Texas MD Anderson Cancer Center, Houston, TX 77030, USA

**Keywords:** STAT3, cancer, therapeutics

## Abstract

**Simple Summary:**

Signal Transducer and Activator of Transcription 3 (STAT3) is involved in many normal cellular processes that are tightly regulated. However, aberrant activation of STAT3 has been implicated in cancer development, recurrence, and metastasis in addition to development of resistance to therapy. Significant progress has been made in targeting STAT3 directly and indirectly through the development of novel therapeutic agents, although some drugs remain in the early phases of development, demonstrating promising potential for future clinical applications. There are several STAT3 inhibitors in clinical trials as monotherapy, and in combination with chemotherapeutic agents and biomarker analysis from these trials, will be critical to inform which patients will benefit from prolonged STAT3 inhibition.

**Abstract:**

Signal Transducer and Activator of Transcription 3 (STAT3) plays a significant role in diverse physiologic processes, including cell proliferation, differentiation, angiogenesis, and survival. STAT3 activation via phosphorylation of tyrosine and serine residues is a complex and tightly regulated process initiated by upstream signaling pathways with ligand binding to receptor and non-receptor-linked kinases. Through downstream deregulation of target genes, aberrations in STAT3 activation are implicated in tumorigenesis, metastasis, and recurrence in multiple cancers. While there have been extensive efforts to develop direct and indirect STAT3 inhibitors using novel drugs as a therapeutic strategy, direct clinical application remains in evolution. In this review, we outline the mechanisms of STAT3 activation, the resulting downstream effects in physiologic and malignant settings, and therapeutic strategies for targeting STAT3. We also summarize the pre-clinical and clinical evidence of novel drug therapies targeting STAT3 and discuss the challenges of establishing their therapeutic efficacy in the current clinical landscape.

## 1. Introduction

**Figure 1:**
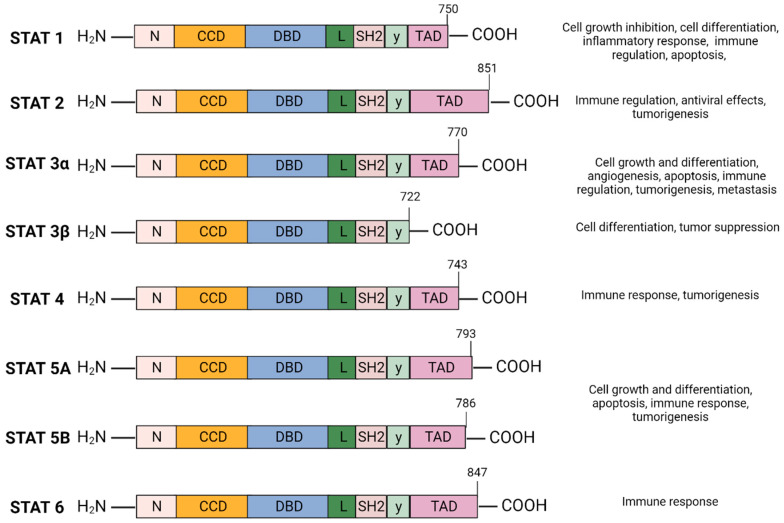
STAT protein family domain structure and function: The STAT protein family has a conserved structure characterized by six domains. N-terminal domain (NTD) facilitates STAT3 DNA promoter binding and assembly of transcriptional machinery. The coiled-coil domain (CCD) promotes STAT3 recruitment to the receptor and facilitates downstream interactions—phosphorylation, dimerization, nuclear translocation. The DNA-binding domain (DBD) is required for STAT3 binding to STAT3-regulated DNA sequence promoter. The linker domain connects the DBD to the SRC-homology-2 (SH2) domain. SH2 domain mediates receptor tyrosine phosphorylation and stabilizes dimerization of the STAT3 protein by interacting with phosphorylated tyrosine residues of a different STAT3 monomer. The carboxyl-terminal transactivation domain (TAD) contains tyrosine residue Tyr705 and Ser727 phosphorylation sites which are essential for the transcriptional activation of target genes. Created with BioRender.com.

The Signal Transducer and Activator of Transcription (STAT) family of cytoplasmic transcription factors is comprised of eight proteins (STAT1, STAT2, STAT3α, STAT3β, STAT4, STAT5A, STAT5B, and STAT6) that share common structural domains and regulate a wide array of crucial cellular processes (Figure 1). They consist of 750–900 amino acids divided into six domains: N-terminal domain (NTD), coiled-coil domain (CCD), DNA-binding domain (DBD), linker domain, SRC-homology-2 (SH2) domain, and the carboxyl-terminal transactivation domain (TAD). STAT proteins facilitate multiple intracellular signaling pathways by transmitting signals from cytokines and growth factors from the plasma membrane to the nucleus, mediating the transcription of downstream gene targets involved in cell proliferation, differentiation and survival, apoptosis, angiogenesis, and immune response (Figure 1) [1,2]. 

STAT1 inhibits cell growth by upregulating cell cycle-related genes including cyclin-dependent kinase inhibitors (p21, and p27) or by downregulating c-Myc. STAT1 also inhibits cell growth by inhibiting the expression of cyclins D2, D3, and E [3]. The majority of STAT2 biological effects are antiviral via interferon stimulation genes. STAT2 Knock Out (KO) mice lack the type I interferon (IFN) autocrine loop and have defective T cell and macrophage response, suggesting STAT2 is critical for immune response regulation [4,5]. Unlike other STATs, STAT4 expression is limited to the bone marrow, thymus, and testes and is crucial for mediating the humoral immune response. STAT4 stimulates maturation and development of B cells and mediates response to interleukin (IL)-12 to induce differentiation of naïve Th0 cells to Th1 cells [6,7]. STAT6 plays an essential role in IL-4 signaling and induces Th2 cell differentiation and immunoglobulin conversion [8,9]. STAT5, originally called prolactin-induced mammary gland factor, is involved in mammary gland development and lactogenesis, which has been studied in STAT5 KO mice found to have defective mammary gland development [10]. STAT5 is also involved in breast tumorigenesis and Janus kinase 1 (JAK1) activation via the prolactin receptor, whereas JAK2 activity has been shown to enhance STAT5 signaling in breast cancer cells [11].

**Figure 2:**
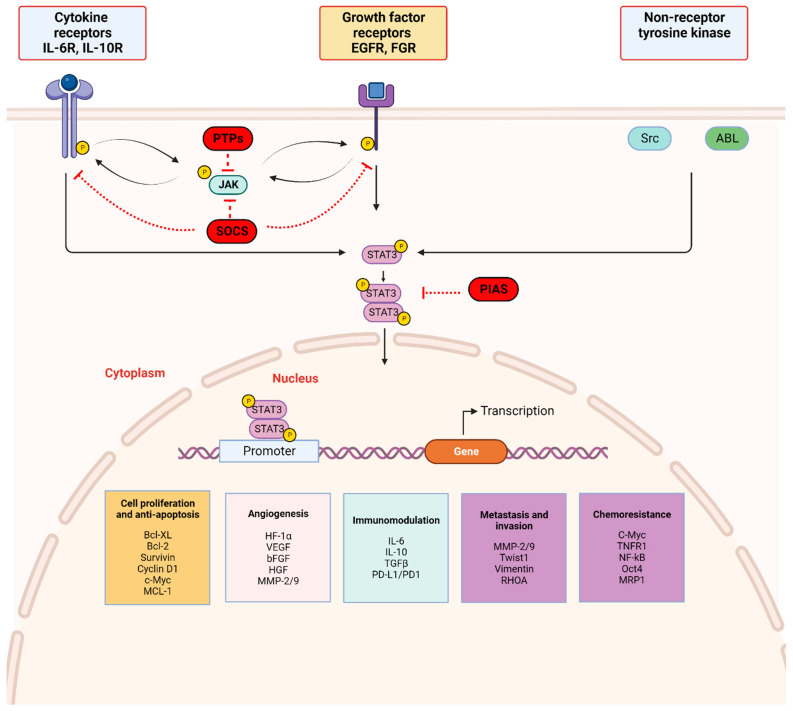
STAT3 signaling pathway: STAT3 activation is initiated by upstream activity including ligand binding by (a) cytokines (IL-6 and non-IL-6 family members), (b) growth factors (EGF, FGF, etc.) to respective receptor-linked kinases, and (c) non-receptor-linked kinases (Scr, ABL). This triggers JAK activation through phosphorylation (p), with subsequent receptor tyrosine phosphorylation and STAT3 phosphorylation at Tyr705. Activated STAT3 forms homodimers which translocate to the nucleus, bind consensus DNA, and regulate the transcription of target genes. Three main endogenous proteins negatively regulate the physiologic activation of the STAT3 signaling pathway; SOCS (suppressor of cytokine signaling), PTPs (protein tyrosine phosphatase), and PIAS (protein inhibitor of activated STAT). SOCS and PTPs interact directly with respective tyrosine kinase receptors and JAK. SOCS block recruitment of STAT3 and inhibit JAK kinase activity while PTPs dephosphorylate related JAK and can directly dephosphorylate STAT3 dimers. PIAS prevents STAT3 dimers from binding DNA. Created with BioRender.com.

STAT3 is the most studied member of the STAT family and, along with STAT5, has been implicated in tumorigenesis [12,13,14,15,16,17]. For instance, STAT3 and STAT5 activation are associated with castration-resistant prostate cancer [15,16]. while in colon cancer, high p-STAT3/p-STAT5 has been shown to predict poor prognosis [17]. STAT3 activation is a tightly regulated process that occurs through phosphorylation of tyrosine and serine residues via signaling from upstream molecules, resulting in transmission of transcriptional signals to the nucleus (Figure 2) [1]. Knock-down of STAT3 in mice is embryonically lethal as shown by the inability of viable STAT3 hemizygous mice to conceive STAT3-deficient mice with no STAT3^−/−^ fetuses identified by E8.5, suggesting that STAT3 has a role in early embryogenesis [18,19]. Because STAT3-deficient mice die early during embryogenesis, a Cre-loxP recombination system was generated to study STAT3 gene ablation later in life in different tissue types [20]. Results revealed that STAT3 is not essential for ex vivo cell viability, as shown with viable STAT3-deficient murine T-cells, mammary epithelial cells, fibroblasts, and macrophages. However, these cells exhibited various degrees of dysfunction [20]. For instance, STAT3-deficient T cells displayed a significantly impaired proliferative response to IL-6, thought to be secondary to a defect in IL-6 mediated suppression of apoptosis, highlighting the anti-apoptotic function of STAT3. In addition, the expression of anti-apoptotic proteins such as Bcl-2, Bcl-XL, Mcl-1, and survivin is induced by STAT3 activation followed by STAT3 binding to DNA response elements [21]. Importantly, the oncogenic effects of STAT3 have been well established, and constitutive activation of the STAT3 signaling pathway is essential for survival in several tumor-derived cell lines, including those derived from multiple myeloma [12], astrocytoma [13], in addition to head and neck cancers [14]. The role of STAT3 in tumorigenesis may also be triggered by enhancing cell cycle progression and inhibiting apoptosis [21]. Studying the oncogenic activity of STAT3 in various cancers provides opportunities for leveraging the STAT3 signaling pathway as a key therapeutic target in cancer treatment. Moreover, several preclinical and clinical studies have generated evidence supporting this therapeutic strategy. 

This review summarizes current knowledge of the mechanisms of STAT3 pathway signaling as elucidated in in vitro and in vivo studies and discusses the role of constitutively activated STAT3 in cancer development. We first review STAT3 activation by multiple ligands including cytokines and growth factors initiated by binding to respective receptor and non-receptor kinases. We also discuss negative regulators of STAT3 activation, such as protein tyrosine phosphatases and suppressors of cytokine signaling. We further describe evidence supporting the overexpression and aberrant activation of STAT3 in different cancers. Finally, we highlight approaches to STAT3 inhibition and novel agents in preclinical and clinical studies targeting STAT3 as a therapeutic strategy for cancer. 

## 2. Mechanism of the STAT3 Signaling Pathway (Figure 1 and Figure 2)

### 2.1. STAT3 Structure and Isoforms (Figure 1)

STAT3, initially called acute phase response factor, was observed to bind IL-6 responsive elements identified in acute-phase protein genes. Akira et al. cloned STAT3 and demonstrated STAT3 activation via tyrosine phosphorylation in the cytoplasm in response to IL-6 and other cytokines with subsequent translocation into the nucleus [22]. The role of STAT3 as a transcription factor was also mediated by the glycoprotein 130 (gp130)-dependent signaling pathway in a variety of cell types [22].

STAT3 has a relative mass of 88 KDa and is comprised of 770 amino acids [23,24,25]. Structurally, STAT3 is similar to other STAT proteins and includes six distinct domains, as depicted in Figure 1. The SH2 domain is highly conserved among the STAT family and is critical for STAT3 dimer formation. It directly interacts with the phosphorylated Tyr705 residue located in the TAD of another STAT3 molecule which induces STAT3 homodimerization. The TAD subsequently promotes transcriptional activation of the target gene (Figure 1) [23,26]. 

STAT3 is located adjacent to the STAT5 locus on chromosome 17, is conserved among species, and has three major isoforms; the α full length form and the shorter γ and β forms generated as a result of mRNA proteolysis and splicing [27]. STAT3α plays a role in cell proliferation, whereas STAT3β and STAT3γ may exert a negative effect on full length STAT3 [28]. Unlike STAT3α, STAT3β lacks the transactivation domain and the 55 amino acids at the C-terminus are replaced by a unique seven-amino acid sequence (Figure 1) [29]. STAT3β primarily regulates cell differentiation, and several studies suggest a significant role in mediating tumor suppression [30,31,32]. STAT3γ may play a role in neutrophil differentiation [31,33]. 

Of the STAT family proteins, STAT3 and STAT5 have generated scientific interest because they are similarly activated by ligands involved in cell proliferation [34], adhesion [35], and angiogenesis [36]. Although their physiologic activation is transient, persistent activation and dysregulation has been implicated in tumor progression, particularly in lymphoma and leukemia [37,38]. They also have been shown to compete for binding sites to regulate B cell lymphoma protein 6 (BCL6), an oncogenic transcriptional modulator. Specifically, STAT3 upregulates BCL6 expression while STAT5 represses BCL6 expression below basal levels in a fashion dominant to STAT3-mediated induction [39].

### 2.2. STAT3 Signaling and Activation (Figure 2)

The JAK/STAT3 signaling axis is a central pathway in cell function and is critical for mediating transduction of cellular physiologic signals involving other signaling pathways, including the phosphoinositide 3 kinase (PI3K), mitogen-activated protein kinase (MAPK), and extracellular receptor kinase (ERK) pathways [40]. Cytokines (IL-6/IL-10) as well as growth factors, including epidermal growth factor (EGF), fibroblast growth factor (FGF), and insulin-like growth factor (IGF), bind corresponding receptor-linked kinases (JAK, tyrosine kinases) and growth factor receptors to trigger a tyrosine phosphorylation cascade. Non-receptor-linked kinases such as SRC and ABL may also initiate this cascade leading to transcription of downstream targets [41,42]. Following ligand binding, the receptors form dimers that recruit glycoprotein 130 (gp130) and JAKs, with subsequent phosphorylation and activation of JAKs. The cytoplasmic tyrosine residues of the receptors are in turn phosphorylated by JAK, prompting their interaction with the STAT3 SH2 domain and subsequent STAT3 phosphorylation at the Tyr705 residue located in the TAD. Phosphorylation of STAT3 induces dimerization of two STAT3 molecules in a tail–tail conformation via reciprocal phosphorylated tyrosine (Tyr705)-SH2 domain interactions. This signaling triggers nuclear translocation of STAT3 in an active process by a group of proteins called importins [43,44]. In the nucleus, activated STAT3 dimers bind to the promoter sequences of target genes via the DBD to initiate transcription of genes involved in cell proliferation, angiogenesis, metastasis, and chemoresistance in addition to suppression of antitumor immunity (Figure 2) [45]. 

STAT3 activation is tightly regulated by endogenous negative modulators, ensuring activation in the normal physiologic state. Three main protein families regulate STAT3 activation: protein tyrosine phosphatases (PTPs), protein inhibitors of activated STAT (PIAS), and suppressors of cytokine signaling (SOCS) (Figure 2) [46]. Activated STAT3 induces SOCS expression, and SOCS proteins act in two main ways: (1) they bind phosphorylated JAK to decrease its kinase activity and (2) they bind phosphotyrosine on receptors to prevent STAT3 recruitment to the receptors [47]. PIAS represents a family of transcriptional receptors that interact with STAT dimers and block STAT3 DNA-binding activity, thereby regulating transcription factors [46,47]. Finally, PTPs such as Src homology domain-containing tyrosine phosphatases 1/2 (SHP-1/2) act by directly dephosphorylating STAT3 or by mediating dephosphorylation of ligand–receptor complexes [47,48,49]. These endogenous inhibitors govern physiologic STAT3 function in normal cells and their activation is a potential therapeutic strategy in cancer cells with activated STAT3.

## 3. STAT3 Activation in Cancer

Aberrant STAT3 phosphorylation is reported in up to 70% of solid and hematologic cancers including multiple myeloma, lymphomas, leukemias, head and neck cancer, and breast cancer [50,51,52]. Many preclinical studies have informed the critical role of STAT3 activation in malignant transformation using tumor-derived cell lines, thus, STAT3 is considered by most to be an oncogene [12,21,53,54]. However, recent studies identified an alternative role for STAT3 as a tumor suppressor in a process dependent on the oncogenic environment and alternative splicing into the STAT3α and STAT3β isoforms. As future research efforts expand their focus on targeting the STAT3 pathway, consideration of the context-dependent role of STAT3 and the corresponding implication in the development of different tumors is essential, as summarized in Table 1. While the oncogenic role of STAT3 is well studied, evidence suggests that STAT3 demonstrates tumor-suppressive functions in certain cancers and this dual role may depend on inherent genomic mutations. In non-small cell lung cancer (NSCLC), aberrant STAT3 activation drives tumor development [55,56]. and increased STAT3 expression correlates with poor outcomes [57]. However, in the context of KRAS mutant NSCLC, compelling evidence suggests that STAT3 is a potent tumor suppressor [58,59]. Knock down of STAT3 in *KRAS* mutant lung adenocarcinoma xenografted cells generated tumors that were more vascularized, had increased growth, and resulted in worse survival [59]. We further expand on evidence supporting the paradoxical role of STAT3 (as an oncogene or as a tumor suppressor gene) in cancer. 

### 3.1. STAT3 and Tumorigenesis

The first study to confirm the oncogenic properties of STAT3 was published in 1995 [53]. In cells transformed by the Src oncogene tyrosine kinase, Yu et al. showed that constitutive STAT3 activation correlated with Src transformation and was associated with enhanced STAT3 DNA binding activity [53]. Subsequent studies demonstrated evidence of direct or indirect STAT3 activation by oncogenic tyrosine kinases (v-Ros and v-Abl) [75,76]. or viral proteins (Epstein–Barr virus and human T lymphotropic virus) [77,78]. To expand upon these findings, Bromberg et al. set out to determine if constitutively activated STAT3 could induce malignant transformation independent of a tyrosine kinase oncogene [21]. To this end, they created a mutant form of STAT3 by engineering a constitutively dimerized STAT3 molecule, STAT3-C, by substituting cysteine residues for specific amino acids in the C-terminal loop within the SH2 domain. The STAT3 mutant protein underwent spontaneous disulfide linkage dimerization and immortalized fibroblasts transfected with this STAT3 mutant expression vector formed colonies in soft agar, whereas wild type STAT3 did not. Furthermore, nude mice injected subcutaneously with 10^6^ cells from STAT3-C clones developed tumors within 2–4 weeks compared to parental cells that did not develop tumors over an 8-week period. Importantly, western blot analysis of the STAT3 mutant tumors showed retention of the tagged STAT3-C protein [21]. 

Since these early experiments, oncogenic effects of STAT3 in tumor growth and survival have been demonstrated through upregulation of anti-apoptotic proteins and proteins involved in cell cycle progression while down regulation of these genes as a result of STAT3 inhibition correlates with apoptosis and growth arrest [77,79]. 

Specifically, STAT3 activation confers resistance to apoptosis, a process through which malignant cells evade cell death by upregulating survivin, Bcl-XL, inhibitors of apoptosis protein family proteins, and Mcl-1 [60,80,81,82]. In U266 myeloma cell lines, blocking STAT3 signaling via a JAK family kinase activity inhibitor (AG490) inhibited Bcl-XL expression and induced apoptosis [12]. Similarly, in an astrocytoma cell line, inhibition of STAT3 expression with STAT3-specific small interfering RNA (siRNA) increased caspase-dependent apoptosis and decreased expression of STAT3 target genes survivin and Bcl-XL [13].

These studies suggest that STAT3 protects transformed cells against apoptosis. STAT3 also exerts effects on the cell cycle by mediating G1 to S phase cell-cycle transition through upregulation of cyclins D1, D2, and D3 and downregulation of p21 and p27 [83,84]. In cell lines expressing an oncogenic STAT3 variant, cyclin D1 mRNA levels were increased promoting retinoblastoma protein (Rb) phosphorylation, thereby leading to E2F transcription factor release, transcription, and S phase entry [83]. This is critical, as cyclin D1 overexpression is a key agent of mammary tumorigenesis [85]. 

STAT3′s involvement in tumorigenesis includes downregulation of anti-tumor immune surveillance [86,87], epithelial-to-mesenchymal transition (EMT) with subsequent metastasis [88,89], and development of drug resistance [90,91,92]. Induction of tissue remodeling factors that promote EMT, a process critical for progression of epithelial tumors, has been directly linked to STAT-mediated induction of Twist gene expression [93]. The Twist gene encodes the Twist-related protein 1 (Twist1) which is a basic helix-loop-helix (bHLH) transcription factor implicated in cell differentiation, and mutational inactivation of the Twist gene results in skull deformations, limb abnormalities, and facial dysmorphism [94]. Twist is also involved in EMT during mesoderm differentiation in *Drosophila* and is associated with a switch from epithelial cadherin (E-cadherin) to neural cadherin (N-cadherin) expression [95]. This loss of E-cadherin mediated cell–cell adhesion occurs frequently during tumor cell invasion and is thought to promote tumor progression and metastasis [96]. Twist gene overexpression occurs in some human cancers such as hepatocellular carcinoma, breast cancer, and pancreatic cancer and is associated with advanced tumor stage and poor prognosis [95,97,98]. In highly invasive breast cancer cell lines MCF-7-I4 and MDA-MB-453-I4 derived from MCF-7 and MDA-MB-453 cell lines, respectively [99], Cheng et al. were able to demonstrate that inhibition of STAT3 with small molecule inhibitor JSI-124 reduced pSTAT3 levels with a marked reduction in Twist protein expression [100]. STAT3 has also been linked to the development of metastasis facilitated by basal membrane and extracellular matrix degradation in addition to amoeboid movement within [101]. STAT3 activates RhoA, an important actin regulator that drives amoeboid movement in many cancer cells, and JAK/STAT3 inhibition decreases migration of malignant cells as evaluated in 3D collagen gels [102]. STAT3 also binds the promoter of matrix metalloproteinases (MMPs) genes, which are endoproteins with activity against extracellular matrix components. By upregulating their expression, STAT3 activation promotes cancer cell invasion, and STAT3 knockdown reduces MMP expression and pancreatic cancer cell invasiveness in mice [103,104]. Finally, activated STAT3 and hypoxia-inducible factor-1α (HIF1α) bind the promoter of vascular endothelial growth factor (VEGF) mediating its transcription [36]. Expression of STAT3-C, an oncogenic mutant form of STAT3 with constitutive activation, has been shown to increase tumor angiogenesis in mice transfected with B16 melanoma cells [105].

STAT3 signaling is involved in antitumor immunity through multifaceted crosstalk between cancer and tumor-infiltrating immune cells in the tumor microenvironment [106]. As such, STAT3 mediates induction of immunosuppressive tumor-derived factors, including IL-10, IL-6, VEGF, and TGFβ which, in a positive feedback loop, amplifies STAT3 activation, generating a pathway for immune evasion by tumors with constitutive STAT3 activation [107]. STAT3 hyperactivation by tumor-derived growth factors is also involved in abnormal differentiation of myeloid cells into mature dendritic cells [107,108]. Thus, these abnormal cells lack the ability to activate CD8 T cells, a critical step in antitumor immune response [107,108,109]. Hence, targeting STAT3 may inhibit immunosuppressive interactions between tumor cells and tumor-infiltrating immune cells. STAT3 also promotes tumor immune evasion by upregulating immune checkpoint proteins [110,111]. For instance, STAT3 mediates programmed death-ligand 1 (PD-L1) upregulation by binding directly to the promoter of PD-L1 in tumor cells. Decreased PD-L1 levels were noted in MDA-MB-231 breast cancer cells with constitutive STAT3 suppression using selective STAT3 inhibitors [109,110,111]. 

Combination therapy with STAT3 inhibition and immune blockade can address the limited response observed in some patients treated with immunotherapy due to the potential of decreasing side effects if a lower dose of immunotherapy is needed for treatment. Combined inhibition of STAT3 and immune checkpoint has shown promise in the pre-clinical setting. In murine models of pancreatic cancer, treatment with JAK-STAT inhibitor Ruxolitinib suppressed tumor growth, decreased PD-L1 expression, and enhanced Cytotoxic T Lymphocyte activation, with the lowest tumor growth observed with combined Ruxolitinb and anti-PD-1 mAb immunotherapy [110]. There are also known gender differences in the efficacy of immunotherapy due to different circulating concentrations of sex steroids and receptors on immune cells that can transcriptionally modulate innate and adaptive immune cells [112]. For instance, elevated testosterone levels in men is associated with decreased circulating inflammatory cytokines such as TNF which may contribute to a more robust response to ant-TNF therapies. Consideration of these variations will need to be evaluated in the design of clinical trials incorporating immunotherapy and STAT3 inhibition [113].

Finally, STAT3 activity has been shown to confer resistance to chemotherapeutic drugs in multiple tumor cell lines and xenograft models. After treatment with increasing concentrations of paclitaxol, 435B metastatic brain cancer cell lines were more resistant to apoptosis compared to parental MDA-MB-435 breast cancer cells. However, when the 435B cells were transfected with a dominant-negative form of STAT3, increased chemotherapy-induced apoptosis was observed [114]. In a nude mouse xenograft model of hepatocellular carcinoma, combination treatment with cisplatin and YC1, a down regulator of STAT3, suppressed tumor growth compared to treatment with cisplatin alone [115]. The combination therapy also induced corresponding down-regulation of pSTAT3, Bcl-xl, Cyclin D1, and survivin and upregulation of caspase 9 and poly (ADP-ribose) polymerase (PARP) cleavage [115]. These alterations of pro- and anti-apoptotic proteins were observed in a dose-dependent fashion. 

### 3.2. STAT3 as a Tumor Suppressor 

Although the role of STAT3 as an oncogene has been well demonstrated, with many cancers harboring constitutively active STAT3 [19,46,60,77,80,81,84], some studies suggest that STAT3 has a context-dependent tumor suppressor role [29,32]. For example, studies reported that the STAT3β isoform mediates the tumor suppressor effects of STAT3 by forming a heterodimer with STAT3α, thereby inhibiting the transactivation of STAT3α [29,32]. The dual role of STAT3 in the development of human cancers was first examined in glioblastomas [116,117]. These studies demonstrated that the differentiation of neural precursor cells (NPCs) into astrocytes involves the STAT3-mediated blockade of proliferative processes followed by cell differentiation [66,116,117,118]. However, STAT3 has also been implicated in the renewal of NPCs, supporting a dual role in glioblastoma development [116,117]. Furthermore, STAT3 activation in gliomas is variable, with Wang et al. noting focally positive nuclear pSTAT3 staining in only 9% (23 of 254) of gliomas evaluated using immunohistochemistry [118]. PTEN is a tumor suppressor protein that may mediate STAT3’s variable role in cancer development [119]. In glioblastoma cells with functional PTEN, AKT is directly inhibited by PTEN with the suppression of STAT3 activation via the FOXO3/LIFRβ gene [66,119]. Conversely, PTEN-deficient tumor cells have constitutive activation of AKT, and STAT3 is inactive in these cells [66,119]. In thyroid cancer, data strongly suggest that STAT3 is involved in growth suppression [67]. Nuclear pSTAT3 was detected in 57% (63 of 110) of papillary thyroid carcinoma (PTC) cases, and pSTAT3 expression was inversely correlated with tumor size and the presence of distant metastasis [67]. Furthermore, by crossing thyroid peroxidase (TPO)-Cre/STAT3^−/−^ mice with BRAF/STAT3^flox/flox^ mice to generate mice with BRAFV600E expression in thyrocytes with or without STAT3, Couto et al. showed that STAT3-deficient mice had more proliferative and larger tumors than did mice with wild type STAT3 [67]. Also, in the colorectal cancer cell line HCT116, STAT3 knockdown resulted in higher Snail1 (Snail Family Transcriptional Repressor 1) expression and greater invasiveness than controls in cell invasion assays [70]. Snail1 is a zinc finger transcription repressor that induces EMT through suppression of E-cadherin, and evidence supports that STAT3 facilitates glycogen synthase kinase (GSK) 3β-mediated degradation of SnaiI1 through GSK3β phosphorylation [70]. 

Specific to breast cancer, STAT3 promotes tumor proliferation and is active in invasive breast cancer biopsies samples but not in benign breast tumors [62]. Notably, pSTAT3 expression in primary tumor biopsy samples obtained from 346 node-negative breast cancer patients showed improved 5 and 20-year survival rates with STAT3 activation [120]. Another study comparing mutations in synchronous/metachronous metastases with primary breast tumors demonstrated additional mutations in metastases, particularly loss of JAK2 and STAT3, suggesting that the JAK/STAT3 pathway may function as a tumor suppressor and explain the improved short and long-term outcomes in these patients [121]. Also, in head and neck squamous cell cancer cell lines (HNSCCs), constitutive activation of STAT3 is driven by TGFα-induced EGFR activation [54]. Similar to patients with breast cancer and glioblastoma, a survival analysis of 70 patients with HNSCCs demonstrated that patients with high nuclear STAT3-expressing tumors by immunohistochemistry had improved progression-free survival and lower risk of death than did patients with lower STAT3-expressing tumors [64]. Of note, the majority of patients had advanced disease (75% with stage III/IV disease), and this disease profile may be independent of STAT3 pro-oncogenic signaling. As such, treatment strategies targeting STAT3 in cancers should not only account for the state of STAT3-activation, but also address the dominant function of STAT3 in tumors. The genomic profile of the tumor may further elucidate if STAT3 performs in an oncogenic or tumor suppressive fashion in each tumor. Notably, novel biomarkers beyond overexpression of STAT3 pY705 may provide a more comprehensive approach to guiding STAT3-directed treatments and may include upstream ligands such as IL-6 that could be serially monitored with the convenience of a blood test compared to serial tumor biopsies.

## 4. Strategies for Targeting STAT3 Signaling

**Figure 3:**
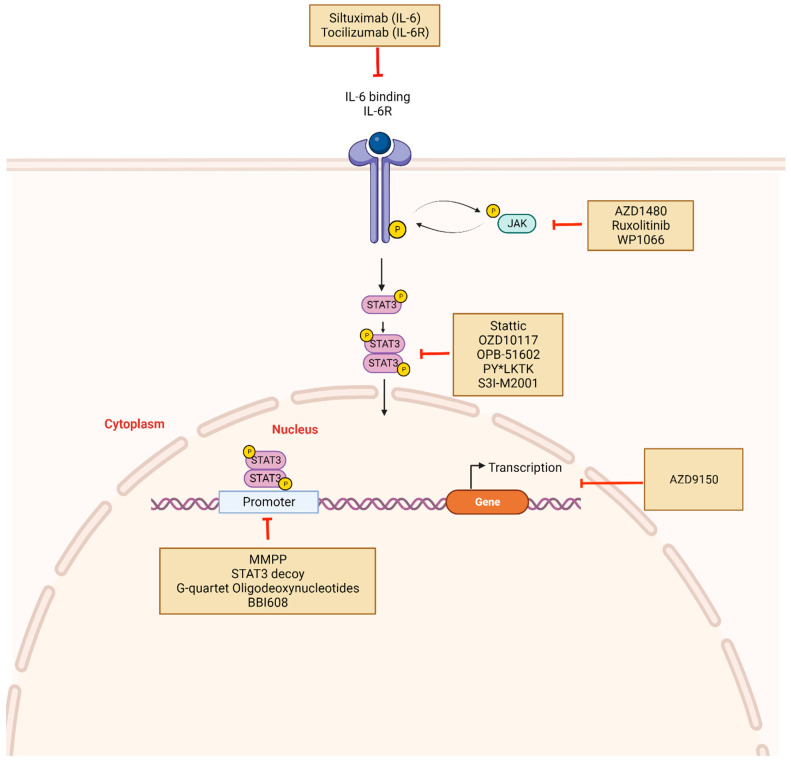
STAT3 inhibitors and mechanism of action: Constitutive activation of STAT3 can be disrupted indirectly or directly using novel drugs. Indirect inhibitors target upstream molecules such as IL-6, IL-6R, or JAK and include Tocilizumab, Siltuximab, Ruxolitinib. Direct inhibitors prevent STAT3 phosphorylation, dimerization, and target gene transcription. These include Stattic, OZD10117, OPB-51602, and PY*LKT. Other mechanisms of STAT3 inhibition involve targeting DNA-binding domains with molecules such as DNA decoy oligonucleotides. STAT3 mRNA may also be targeted using antisense oligonucleotides such as AZD9150. Created with BioRender.com.

Considering the role of STAT3 activation in the development of various cancers, STAT3 inhibition has been extensively studied as a viable target for cancer therapy, with considerable effort directed toward translating preclinical findings into the clinical trial setting. (Table 2) These ongoing clinical trials are designed to evaluate STAT3-targeted strategies as single agents or in combination with chemotherapeutic agents to overcome resistance to standard therapy. As depicted in Figure 3, two main approaches in achieving STAT3 inhibition used are 1) indirect inhibitors that target elements of the STAT3 signaling pathway, including JAK and IL-6/IL-6R and 2) direct inhibitors that directly block the activity of the STAT3 domains—SH2, DBD, and NTD. Because indirect STAT3 inhibitors lack specificity, directly inhibiting STAT3 is viewed as a more appealing approach. However, the success of direct inhibitors has been limited and the STAT3 protein has been described as “undruggable”, a term referring to a protein incapable of direct pharmacologic targeting. Other examples of undruggable targets are the well-studied P53 tumor suppressor protein and MYC proto-oncogene [122]. Furthermore, the STAT3 protein–protein interaction involves a large and flat surface area that may limit selective inhibitor binding compared with binding pockets in other proteins such as receptor tyrosine kinases [123]. Furthermore, STAT family proteins have highly homologous domains, making STAT3-specific targeting challenging. Several other inhibitors targeting STAT3 in multiple cancer types are currently in clinical trials.

### 4.1. Indirect Inhibitors of STAT3 

Targeting upstream/downstream components of the STAT3 signaling pathway is a competitive strategy for blocking STAT3 activation. Some upstream targets include JAK-associated cytokine receptors, IL-6/IL-6R, and non-receptor kinases such as Src and Abl. Because indirect STAT3 inhibitors lack specificity for STAT3, the compounds may cause broad kinase inhibition, and off-target effects in the clinical setting continue to represent a major concern (Table 2) [124]. 

JAK inhibitors have been investigated in several clinical trials for the treatment of solid tumors. AZD1480 is a small molecule JAK1/2 inhibitor that inhibits constitutive JAK/STAT in the colorectal cancer cell lines HCT116, SW480 and HT29 [125]. In a study by Stuart et al., AZD1480 therapy successfully suppressed inflammation-associated gastrointestinal cancer progression in vivo [126]. Furthermore, Sen et al. demonstrated abrogation of STAT3 phosphorylation and anti-tumor activity in human papillomavirus-HNSCC mouse models and in patient-derived HNSCC xenograft models [14]. In spite of these promising results, dose-limiting toxicities (DLTs) limit the clinical application. This was demonstrated in a phase 1 clinical trial of 38 patients with advanced malignancies treated with AZD1480 which had limited clinical activity but with significant DLTs [127]. 

Ruxolitinib is an orally bioavailable JAK1/2 inhibitor that functions by inhibiting STAT3 phosphorylation and is approved by the U.S. Food and Drug Administration (FDA) for treating steroid refractory graft-versus-host-disease [128,129], myelofibrosis [130], and polycythemia vera [130]. In pre-clinical studies of myeloproliferative neoplasms, Ruxolitinib inhibited the proliferation of JAK2 V617F-driven Ba/F3 cells that constitutively phosphorylate JAK2 [131]. Authors noted a dose-dependent decrease in phosphorylated JAK2 and STAT5 but no changes in the total levels of the proteins [131]. Similarly, the authors observed reduction in phosphorylation of STAT3 and STAT5 in human HEL cell lines with endogenous JAK2V617F expression [131]. In this study, while pSTAT3 and pSTAT5 were completely absent in cells at highest treatment doses, HEL cell proliferation was not completely inhibited, suggesting that cell growth was not entirely dependent on JAK2V617F [131]. In JAK2V617F^+^ cells from polycythemia vera patients, dose-dependent reductions in erythroid and myeloid progenitors were observed with Ruxolitinib use. Furthermore, in a murine model of JAK2V617F-driven cancer generated by injecting mice with JAK2V617F-expressing hematopoietic cells, treatment with Ruxolitinib decreased severity of splenomegaly and increased survival rates with suppression of cytokine signaling as shown by a corresponding decrease in proinflammatory cytokines including IL-6 and TNF- α [131]. In vitro evidence supporting that Ruxolitinib induced decreases in cytokine levels and disruption of STAT3 activation via upstream signaling suggests a role for Ruxolitinib in cancer cases characterized by elevated IL-6 levels [132,133,134,135,136]. For instance, studies identified higher levels of serum IL-6 in breast cancer patients compared to healthy individuals [132]. as well as association with aggressive disease and poor outcomes [133,134]. Other investigators discovered associations of high serum IL-6 levels with poor response to therapy, including resistance to chemotherapy and endocrine therapy, providing the rationale for combination treatment of breast cancer therapy with Ruxolitinib [135,136,137]. Most recently, combination treatment with aromatase inhibitor in aromatase inhibitor refractory hormone receptor-positive metastatic breast cancer in 21 patients demonstrated on-target inhibition of STAT3 phosphorylation [138]. However, the impact on tumor growth was minimal [138]. When inflammatory biomarkers were examined, no difference were found in the changes in IL-6 and CRP from baseline through treatment between responders and non-responders to therapy [138]. This demonstrates some of the challenges with re-capitulating preclinical study findings in human clinical trials. Improving robustness of preclinical models by using novel systems that are similar to the human tumor microenvironment and early efforts to identify biomarkers of response will aid in translating preclinical evidence into tangible advances in patient care. 

WP1066 is another small molecule inhibitor of JAK2 phosphorylation that is synthesized through structural modification of the precursor small molecule STAT3 inhibitor AG490 as shown by Priebe and colleagues at the University of Texas MD Anderson Cancer Center [139]. Unlike its precursor, WP1066’s solubility enables its transit through the blood brain barrier, and the favorable protein–kinase inhibitory profile makes WP1066 a good candidate for clinical use [140]. In vitro, WP1066 demonstrated potent antitumor effects in U87-MG and U373-MG malignant glioma cells [141]. Treated cells also showed dose-dependent inhibition of STAT3 phosphorylation with decreased detection of nuclear pSTAT3 [141]. The percentage of transferase-mediated dUTP nick-end labeling (TUNEL)-stained cells also increased after treatment with WP1066. Western blot analysis of cell lysates showed downregulation of the antiapoptotic proteins Bcl-XL, Mcl-1, and c-Myc [141]. In a murine subcutaneous model of malignant glioma, intraperitoneal injections of WP1066 inhibited the growth of tumors generated from U87-MG cells. WP1066 has been effective against bladder cancer [142], Acute Myelogenous Leukemia (AML) [139], and melanoma [143], with improved chemo-sensitivity suggested in a variety of tumor models including oral squamous cell carcinoma models [142]. 

Itacitinib is a potent orally selective JAK1 inhibitor with promising preliminary effectiveness in a phase 1 study including patients with corticosteroid refractory or corticosteroid naïve Graft-versus-host disease (GVHD) [144]. The GRAVITAS-301 international double-blind phase 3 study of Itacitinib in combination with corticosteroids demonstrated that Itacitinib was well tolerated but the objective response rate (ORR) at day 28 did not reach prespecified significance levels compared to placebo [145]. Currently, a phase 1 clinical trial aims to establish efficacy in patients with sarcoma and advanced hepatocellular carcinoma (Table 2). Pacritinib is an oral JAK2 inhibitor currently approved by the FDA for treatment of intermediate- or high-risk primary or secondary myelofibrosis [146]. Encouraging data from the phase 3 PERSIST-2 trial demonstrated reductions in spleen volume of at least 35% in 29% of patients receiving Pacritinib but only in 3% of patients receiving best available therapy, including Ruxolitinib [147]. 

Because IL-6 is an upstream target of STAT3 and elevated IL-6 levels in cancer patients have prognostic significance regardless of the cancer type [148], several compounds targeting IL-6 or IL-6R have been developed and are used for both non-malignant and malignant diseases [149,150]. Siltuximab, a monoclonal antibody that functions by preventing IL-6 binding to IL-6 receptors, was the first FDA-approved anti-IL-6 antibody [149]. It is currently approved for the treatment of Multicentric Castleman’s disease which is characterized by hyperplasia of lymphoid tissue [151]. A phase 1/2 dose escalation study of Siltuximab in patients with advanced solid tumors demonstrated that the drug was well tolerated in patients with colorectal, ovarian, and pancreatic cancer [152]. In the phase 1 dose escalation cohort, Siltuximab was administered intravenously at different doses (2.8–15.0 mg/kg) every 2–3 weeks. Patients in the phase 1 expansion/phase 2 cohort received the recommended phase 2 dose of 15 mg/kg every 3 weeks [152]. No objective response was observed in the 84 patients included in the study and 5 of the patients had stable disease for more than 6 weeks [152]. Of note, serum biomarker analysis demonstrated a greater than 50% decrease in CRP levels from baseline in a dose-dependent fashion that was sustained throughout treatment [152]. Of the 84 patients in the cohort, 48 (57%) had measurable IL-6 serum concentrations using an assay that detects all forms of IL-6, and 47 (55%) had elevated baseline IL-6 values (≥10 pg/mL) [152]. However, serum concentrations of IL-6 remained stable during treatment, and no consistent changes in IL-6 RNA expression was observed in 28 patients with paired pre- and posttreatment blood samples. Reduced pSTAT3 levels were observed in 13 patients with biopsy samples [152]. Given the lack of clinically relevant responses in this cohort of patients with advanced solid tumors, the authors hypothesized that late-stage disease may not be IL-6 dependent or that IL-6’s effects are multifactorial, including a robust autocrine effect in that setting which supports consideration of a multimodal approach instead of single agent IL-6 blockade [153].

Tocilizumab is a humanized monoclonal antibody targeting IL-6R and its therapeutic uses include various inflammatory conditions such as rheumatoid arthritis, giant cell arthritis, and cytokine release syndrome [150]. Using tocilizumab, Shinriki et al. evaluated inhibition of IL-6R in a xenograft model inoculated with an oral squamous cell carcinoma (OSCC) cell line (SAS) and observed marked tumor growth inhibition, which did not occur in vehicle-treated controls [154]. pSTAT3 evaluation via IHC demonstrated reduced STAT3 phosphorylation without altering total STAT3 expression [154]. Preclinical evidence suggests that combination treatment of standard of care with Tocilizumab can have significant anti-tumor effects when standard therapy fails. In HNSCC tumors, despite high EGFR expression [155], EGFR tyrosine kinase inhibitors such as erlotinib have had limited effectiveness against HNSCC [156]. As such, Stanam et al. investigated mechanisms of resistance and treatment strategies in HNSCC [157]. They found that IL-6 was significantly upregulated in erlotinib-resistant HNSCC cells more so than in erlotinib-sensitive parental cell lines using gene expression profiling, reverse transcription–polymerase chain reaction (RT-PCR) and enzyme-linked immunosorbent assay (ELISA) [157]. Also, in SQ20B xenograft tumor models, treatment with Tocilizumab and erlotinib resulted in significantly decreased tumor growth and improved survival than in mice treated with erlotinib alone (median survival 33 days vs. 23 days) [157].

Targeting non-receptor tyrosine kinases to indirectly suppress STAT3 activity has also been investigated [158,159]. Dasatinib, an Src inhibitor, is one of four tyrosine kinase inhibitors (TKIs) approved by the FDA for the frontline treatment of chronic myeloid leukemia [160,161]. Similar to other FDA-approved TKIs (Imatinib, Nilotinib and Busotinib), Dasatinib also inhibits Src family kinases, but it is more potent with improved outcomes in the second line setting [158]. Unfortunately, limited effectiveness and significant toxicity in cancer treatment trials have restricted the use of Dasatinib and other Src inhibitors for cancer treatment [159].

### 4.2. Direct Inhibitors of STAT3: Targeting the Various STAT3 Domain Structures to Block the Transcriptional Activity of STAT3 (Figure 3) 

As a transcription factor, STAT3 forms protein complexes that are directed to DNA-specific sites, thereby regulating oncogenic actions [46]. Owing in part to similarities with other members of the STAT family, poor bioavailability, and significant toxicity, direct inhibition of STAT3 has been challenging [162,163]. Direct inhibitors of STAT3 prevent its dimerization by targeting and binding to functional STAT3 domains, specifically SH2, DBD, and NTD [46]. These inhibitors are generally classified as small molecules, peptides, or oligonucleotides (Figure 3). Many small molecule inhibitors have been developed, and although they produced excellent outcomes in vitro (as detailed below for each class of inhibitor), several of them translate poorly into the clinical setting, which may be secondary to low cell permeability (Figure 3 and Table 2) [162]. 

#### 4.2.1. Inhibitors of the SH2 Domain

Ligand-induced tyrosine phosphorylation of gp130 transmembrane receptor by JAKs induces recruitment and binding of SH2 domain-containing signaling molecules such as STAT3, and interactions between the SH2 domain and phosphorylated tyrosine residues are responsible for subsequent protein–protein interactions, including STAT3 phosphorylation [164]. The SH2 domain also facilitates dimerization of STAT3 monomers via interactions between the SH2 located on one monomer and the pY-peptide on the other (Figure 2) [165]. The pY-peptide binding site located on the SH2 domain has been well characterized via docking screens and subsequently identified as a target site amenable to small molecule inhibition [165]. An X-ray crystal structure of STAT3 revealed three specific proximal binding subpockets suitable for targeting by small molecules [165]. Inhibitors of STAT3 that target the SH2 binding domain are designed to compete with the p-Tyr705 binding at this location, and screening strategies such as structure-based virtual ligand screens and cell-based phenotypic screening have been used to identify the optimal SH2 domain drug [166]. As such, they function by blocking recruitment of activated receptor tyrosine kinases and non-receptor kinases that phosphorylate STAT3, as well as by preventing dimerization of two activated STAT3 molecules which disrupts the STAT3 heterodimer-DNA complex [165]. 

Peptides and peptidomimetics constitute a large proportion of SH2 inhibitors, and the first in this class is PY*LKTK (Figure 3) [167]. Through systematic analysis of the SH2-binding peptide, Turkson et al. developed the synthetic peptide PY*LKTK with the ability to inhibit STAT3 activity in vitro [167]. Compared to control, PY*LKTK resulted in dose-dependent reduction in STAT3 DNA binding in nuclear extracts containing active STAT3 [167]. Also, in Src-transformed fibroblasts with constitutively active STAT3, treatment with PY*LKTK resulted in markedly reduced STAT3 DNA binding than in extracts prepared from non-treated cells [167]. 

Application of structure-based computational methods has identified other peptidomimetic molecules such as S3I-M2001, an oxazole-based peptidomimetic that selectively disrupts STAT3 dimers (Figure 3) [168]. In the NIH3T3/v-Src and MDA-MB-435 human breast cancer cell lines that harbor constitutively active STAT3, S3I-M2001 treatment inhibited STAT3-dependent transcriptional regulation of several tumor survival genes, including Bcl-XL and suppressed the growth of human MDA-MB-231 breast tumor xenografts [168]. In addition, TTI-101, formerly known as C188-9, is a competitive inhibitor of STAT3 designed by Tweardy et al. in collaboration with Tvardi Therapeutics [169]. By binding the pY-peptide binding site in the SH2 domain, TTI-101 restricts STAT3 migration to activated cytokine receptor complexes, inhibits STAT3 tyrosine phosphorylation, and hinders STAT3 function [44,169,170,171]. In vitro studies of TTI-101 conducted with the human hepatocellular carcinoma (HCC) cell lines (Huh7, PLC/PRF/5, and HepG2) demonstrated cell growth inhibition [172]. In mice with hepatocyte-specific deletion of *Pten* (Hep*Pten*^−^), TTI-101 inhibited tumor growth and reduced tumor development [172]. In nude mice bearing radioresistant head and neck squamous cell carcinoma xenografts of UM-SCC-17B, TT1-101 prevented tumor xenograft growth. Furthermore, the drug exhibited acceptable oral bioavailability, high concentrations at tumor sites, and limited toxicity [169]. A first-in-human phase 1 dose escalation trial by Tsimberidou et al. was conducted in patients with advanced/refractory solid tumors who had experienced standard therapy failure [173]. In the study, patients received twice daily TTI-101 using a 3 + 3 dose escalation design. No DLTs were observed, and of 39 patients evaluable for response, 5 (13%) had partial responses and 16 (41%) had stable disease. In addition, of 15 patients with HCC, 3 (20%) had confirmed partial response (cPR) with a median duration of 10.5 months. Confirmed partial responses were also noted for ovarian and gastric cancers [173]. These promising results are being further investigated in multiple trials [174,175,176]. Investigators in the multicenter phase 2 REVERT-Liver cancer clinical trial will evaluate the safety and effectiveness of TTI-101 in patients with histologically or radiologically confirmed locally advanced HCC with measurable disease according to the Response Evaluation Criteria in Solid Tumors (RECIST) criteria. (NCT05440708) In three study arms, patients will be given (1) TTI-101 monotherapy, (2) TTI-101 in combination with pembrolizumab, and (3) TTI-101 in combination with atezolizumab and bevacizumab. The study end points include the incidence of adverse events and overall response rates [174].

In breast cancer cases, STAT3 has been implicated in the development of resistance to cyclin-dependent kinase 4/6 inhibitors such as Palbociclib [92]. In preclinical studies, Palbociclib-resistant breast cancer cells demonstrated upregulation of the IL-6/STAT3 pathway, and treatment with TTI-101 significantly increased cell death [92]. A phase 1b/2 clinical trial evaluating TTI-101 in combination with Palbociclib and aromatase inhibitor therapy for progressive metastatic hormone receptor-positive HER2-negative breast cancer (NCT05384119) is underway [175]. STAT3-SH2/pY-peptide interaction is also a potential target for non-peptide small molecules identified via high-throughput screening of chemical libraries. Stattic, also known as STAT3 inhibitory compound, disrupted STAT3 pY-peptide interaction and inhibited IL-6-induced STAT3 activation, nuclear accumulation, and DNA-binding activity in preclinical studies [177,178,179]. Stattic has also induced growth inhibition in several cancer cell lines and the human laryngeal squamous cell UM-SCC-17B xenografts and stattic induced apoptosis in STAT3-dependent MDA-MB-435S breast cancer cells (Figure 3) [179].

Silibinin, the main component of Silymarin extract from herb milk thistle, has been studied as a natural small molecule down-modulator of STAT3 activity [180]. In the DU145 human prostate cancer cell line, Silibinin decreased activated STAT3 in a dose-dependent manner [181]. In the MGC803 gastric cell line, Silibinin inhibited cell growth, and induced apoptosis with corresponding cell arrest in the G2/M phase of the cell cycle [182]. A study by Priego et al. using the Silibinin-based nutraceutical Legasil^®^ demonstrated significant improvement in heavily pretreated patients with brain metastasis with improvement in overall survival compared to controls (15.5 vs. 4 months) [183]. Enhanced bioavailability of the formulation of Silibinin using a phytolipid delivery system was thought to improve Silibinin’s blood–brain barrier permeability [184,185]. It is now currently in a phase 2 trial for NSCLC and breast cancer patients with single brain metastases. (NCT05689619) 

Pyrimethamine is an antiparasitic drug approved by the FDA for treatment of toxoplasmosis. Treatment of Chronic Lymphocytic Lymphoma (CLL) cells with pyrimethamine was shown to decrease expression of STAT3 signature genes (AIM2, ATXN1, ENPP2, GAB1, and ID3) that previously exhibited higher expression in CLL cells compare to healthy B lymphocytes [186]. Furthermore, in a phase 2 trial in which 16 heavily pretreated CLL patients were treated with Pyrimethamine, no DLTs were observed, and 50% of patients had stable disease. Assessment of the expression levels of the five STAT3 signature genes through treatment compared to baseline demonstrated initial decreased STAT3 gene expression with an increase in gene expression noted at time of progression [186].

#### 4.2.2. Inhibitors of the DNA Binding Domain 

The STAT3 DBD binds DNA elements within promoter sites to mediate transcription of related genes and efforts targeting the interactions of STAT3 DBD have been explored [45,46]. Decoy oligonucleotides are a treatment strategy in which the double stranded DNA decoy oligonucleotides imitate elements of the STAT3 transcription factor and competitively bind STAT3, preventing binding to promoter elements of target genes [187]. This results in downstream inhibition of transcriptional activity in the cell [187]. While decoy oligonucleotides have successfully decreased the STAT3-induced transcriptional gene expression in a variety of cancers including head and neck [188], ovarian [189], prostate [190], and hepatocellular carcinoma [191], in vivo application has been limited by plasma instability [192]. To address this, the double strands of the linear STAT3 decoy were linked using hexaethylene glycol spacers to generate a cyclic STAT3 decoy, which has demonstrated increased thermal and nuclease stability [193].

BBI608 (Napabucasin), is another small molecule inhibitor that selectively binds to the DBD of STAT3 (Figure 3) [194]. In vitro studies demonstrated that BBI608 effectively blocked cancer relapse and metastasis in pancreatic cancer xenograft mice who developed tumors after treatment with gemcitabine [194]. Tumors from the treated mice were collected and self-renewal capacity was assessed as a surrogate for stemness through their ability to grow as spheres [194]. In addition, BBI608-treated cells had five-fold decreased stemness compared to gemcitabine-treated cells. A dose-dependent decrease in genes implicated in cancer stem cell self-renewal such as Nanog, Axl, Sox-2, Klf4, survivin, c-Myc, Bmi-1, and β-catenin protein was also noted with BBI608 therapy [194]. After a phase 1b/2 trial of BBI608 combined with panitumumab in KRAS wild-type patients with metastatic colorectal cancer demonstrated promising activity, BBI608 was advanced to a double-blind randomized multicenter phase 3 trial with impressive outcomes as monotherapy in advanced colorectal cancer patients who had received first-line therapy [195]. Patients were randomized to receive twice daily BBI608 at 480 mg compared with placebo and stratified according to KRAS status, prior VEGF inhibitor treatment and time from diagnosis of metastatic disease (<18 months vs. ≥18 months). The primary endpoint, overall survival, did not differ between both groups and neither did progression-free survival [195]. However, the preplanned biomarker analysis of pSTAT3 expression by IHC demonstrated pSTAT3 as a prognostic marker, which was also predictive of benefit from BBI608 therapy. Specifically, of 251 patients who underwent biomarker analysis, 22% had pSTAT3-positive tumors and had significantly worse overall survival compared to patients with pSTAT3-negative tumors. Moreover, in the pSTAT3-positive patient group, overall survival was improved with BBI608 therapy compared to placebo (5.1 months vs. 3.0 months) [195]. 

#### 4.2.3. Inhibitors of the N-Terminal Domain (NTD) 

The NTD directs STAT3-mediated transcriptional activity including STAT3 dimer binding to DNA, unphosphorylated STAT3 dimerization [196], STAT3 tetramer formation from assembled phosphorylated STAT3 Y705-dimers [197], and interactions with proteins to induce chromatin structure changes (Figure 1) [198] The NTD also plays a role in the nuclear translocation of STAT3 as a result of STAT3 interaction with peptide hormones [196,199]. There has been interest in developing compounds that interact and inhibit activity of this domain. In preclinical trials, ST3-Hel2A effectively inhibited the STAT3 NTD and blockaedSTAT3 dimerization in DU145 prostate cancer cell lines [200]. Furthermore, upregulation of proapoptotic genes were observed after ST3-Hel2A therapy in comparison to a control peptide. The induction of proapoptotic genes was also observed in breast cancer cell lines (MCF-7) and but not in normal epithelial cells MCF-10A, HMEC, and RWPE-1 [200].

### 4.3. Other Mechanisms of STAT3 Inhibition

Endogenous STAT3 regulators control activated STAT3 levels by PIAS protein interactions, protein phosphatase receptor complex dephosphorylation, and JAKs degradation by the SOCS protein family (Figure 2) [46]. In cancer cells, one of the mechanisms by which constitutive STAT3 activation is initiated is by downregulation of endogenous regulators such as PTPs, PIAS, and SOCS [46]. For instance, Src homology region 2 (SH2) domain-containing phosphatase 1 (Shp1) is a non-receptor tyrosine that acts as a tumor suppressor as part of the PTP family and is altered in solid and hematologic cancers with significant impact on signal transduction in cancer pathogenesis and progression [201]. In STAT3 signaling, Shp1 directly dephosphorylates the Tyr705 phosphorylation site on STAT3 and as such, has been harnessed as a strategy for modulating STAT3 activity with the aim of inhibiting tumor growth and preventing development of therapy resistance [202,203,204]. Dovitinib (TKI258) is a small molecule inhibitor of fibroblast growth factor receptor 1 (FGFR1), FGFR2, and FGFR3 that has demonstrated cell growth inhibition in FGFR-amplified breast cancer cell lines and xenograft models (HBCx2) [205]. These associated anti-tumor effects are also enhanced by binding directly to Shp1, thereby promoting Shp1 phosphatase activity that involves dephosphorylating JAK kinases and STAT3 directly [201,206]. In a phase 2 clinical trial of postmenopausal patients with hormone receptor positive, HER2-negative advanced breast cancer, dovitinib combined with fulvestrant prolonged progression-free survival in patients with FGF amplification (10.9 vs. 5.5 months) [207]. The study was terminated early due to slow enrollment and no new safety concerns were identified with dovitinib use [207].

Several in vitro and in vivo studies have explored the utility of small interfering RNAs (siRNAs) and short hairpin RNA (shRNA)-based gene-silencing strategies in STAT3 [208,209,210,211]. RNA interference (RNAi) is a naturally occurring post-transcriptional mechanism for silencing genes initiated by double-stranded RNA (dsRNA) not typically found in cell cytoplasm [212]. The dsRNA are subsequently cleaved by dicer endonucleases into 20–25 nucleotide dsRNA known as siRNAs [212]. After strand separation by RNA-induced silencing complex, the single strand guides sequence-specific degradation of homologous RNA [212]. This gene-silencing strategy has shown promise in STAT3 targeting. In particular, STAT3 silencing by siRNAs delivered by aptamer delivery agents have the unique advantage of being small and stable with low immunogenicity [213]. In glioblastoma, a novel aptamer-siRNA chimera (Gint4.T-STAT3) decreased cell viability in vitro and demonstrated anti-tumor effects in in vivo subcutaneous xenograft mouse models [214]. In this study, U87MG glioblastoma cells were injected in athymic nude mice and tumor-bearing mice were treated with intraperitoneal Gint4.T-STAT3. The treatment group induced more significant tumor growth reduction compared to placebo. IHC assessment of the tumors showed higher proliferation in the tumors as measured by the nuclear antigen ki-67 compared to treated tumors (50% vs. 25%) [214]. Key challenges included poor membrane permeability and decreased stability requiring frequent drug delivery [215]. A double-stranded shRNA strategy was developed to address this drawback. Using DNA-directed RNA, shRNA is expressed in cells following insertion of a DNA construct into the nucleus. For as long as a cell continues to produce its own shRNA, the gene silencing persists [212].

The extensive cross talk and alternative signaling pathways that impact STAT3 signaling suggest that single agent inhibitors may not be as effective, and several clinical trials are evaluating STAT3 inhibitors in combination with other therapeutic agents such as chemotherapy in efforts to overcome therapy resistance (Table 2). The rationale supporting this approach is based on STAT3 signaling activity that drives resistance through apoptosis [114], and further evidence that STAT3 inhibition may reverse apoptosis-mediated therapy resistance while re-sensitizing tumor cells to therapeutic agents [216,217]. One example is in B-cell lymphoma [218]. Because B-cell lymphoma is a heterogenous disease with many genetic subtypes which correlate with response to certain therapies, a PRISM master protocol was developed to evaluate multiple targeted therapies alone or in combination with existing therapy for treatment of relapsed or refractory aggressive disease [218]. The Bruton tyrosine kinase (BTK) inhibitor Acalabrutinib has shown some activity in refractory B-cell lymphoma but with limited durable response and a phase 1 trial is currently evaluating combination with AZD9150 as part of the PRISM study [219]. AZD9150 is a second-generation antisense oligonucleotide targeting the 3′-untranslated region (3′-UTR) of the STAT3 gene with reduction in STAT3 expression noted in a variety of preclinical cancer models [220,221,222]. In a phase 1b clinical trial of 30 pretreated patients with B-cell lymphoma, AZD9150 was well tolerated at doses of 2 or 3 mg/kg weekly [223]. Clinical response was observed in 13% of patients and in patients with a complete response, response was durable [223]. There are also ongoing trials evaluating AZD9150 in Non-Hodgkin’s Lymphoma (NHL) and advanced solid tumors [224,225].

Fedratinib targets cells with JAK2 mutations commonly found in hematologic cancers [226]. Ivosidenib and enasidenib are respective isocitrate dehydrogenase enzyme (IDH)1 and IDH2 inhibitors that are used for treatment of accelerated or blast myeloproliferative neoplasm patients with targetable mutations in IDH1/IDH2 [227]. It is thought that combination therapy using Fedratinib and Ivosidenib/enasidenib may be more effective in advanced hematologic malignancies with an IDH mutation and a phase 1 clinical trial is currently ongoing (NCT04955938) [227]. The JAK 1/2 inhibitor Ruxolitinib is approved for treatment of myeloproliferative disorders with broad anti-inflammatory activity against the cytokine storm often associated with this disease [131]. Ruxolitinib in combination with Decitabine (NCT04282187) and other agents such as Pelabresib (NCT04603495), Carfilzomib (NCT03773107), and CPX-351 (NCT03878199) are currently in clinical trials for myeloproliferative neoplasms (Table 2). For instance, in vitro and murine models have demonstrated synergistic anti-clonal activity of Decitabine, a hypomethylating agent in combination with Ruxolitinib [228]. Mice transplanted with Tp53-KO/JAK2V617F leukemic cells and treated with Ruxolitinib and Decitabine showed significant reduction in spleen and liver weights compared to control. Bone marrow evaluation revealed homogenous expansion of blasts in mice treated with vehicle while mice who received combination therapy showed myeloid maturation [228]. 

## 5. Current Challenges and Future Direction

Despite extensive investigation of STAT3 inhibitors in pre-clinical models with promising antitumor activity, clinical trial results show limited clinical activity and many agents have not progressed beyond the early phases of drug development. A majority of the limitations in clinical translation can be attributed to the challenges of generating highly selective STAT3 inhibitors, and the preserved transcriptional activity of monomeric and unphosphorylated STAT3 [229]. Furthermore, many STAT3 inhibitors with clinical trial-level data target upstream components of the STAT3 pathway such as the IL-6 and JAK and thus, dose-limiting toxicities due to off-target effects of STAT3 inhibitors clearly impact clinical effectiveness and utility. Significant technological advances in drug development have led to discoveries of unique therapeutic options including multiple STAT3 inhibitors currently in clinical trial, however, discovery of novel agents that are effective, demonstrate adequate plasma stability, and have low toxicity profiles are needed. One example is TTI-101, a novel orally delivered small molecule inhibitor of STAT3 utilized in clinical trials initiated to address STAT3-driven disease in hepatocellular carcinoma (NCT05440708) and metastatic breast cancer (NCT05384119). Monotherapy with TTI-101 is tolerable and no DLTs were observed in patients with advanced solid tumors [173]. In metastatic hormone receptor-positive and HER2-negative breast cancer, the currently enrolling single arm phase Ib/II dose escalation clinical trial combines TTI-101 with standard of care palbociclib and aromatase inhibitor upon progression of disease. The trial was designed based on preclinical evidence demonstrating upregulation of IL6/STAT3 pathway as a mechanism of resistance in disease progression and proven STAT3 efficacy in vivo and in vitro. The trial examines endpoints of safety and efficacy with pharmacokinetic and pharmacodynamic studies, in addition to clinical outcomes of PFS and OS. Extensive biomarker analyses will be performed to evaluate association between biomarkers such as serial IL-6 measurements and antitumor efficacy as well as clinical outcomes. 

Key evidence has emerged demonstrating that the dual role of STAT3 depends on the tumor profile and associated mutational changes. As such, determination of the oncogenic or tumor suppressive function of STAT3 should inform the decision to employ STAT3 inhibition as a therapeutic strategy. In order to refine translational efforts aimed at improving the efficacy of novel STAT3 inhibitors in clinical trials, a biomarker-driven approach can help tailor anti-STAT3 treatment and improve therapeutic efficacy of STAT3-targeted agents. Extensive biomarker testing conducted in the TTI-101 clinical trial will be critical to inform personalization of future studies investigating STAT3 inhibition in tumors with oncogenic STAT3 activity. While constitutive activation of STAT3 has been determined by overexpression of STAT3 pY705, more novel methods of accurately identifying patients who may benefit from STAT3 inhibition are needed. Ideally, this approach would incorporate serial monitoring through duration of therapy in order to identify a subset of cancer patients who will benefit from prolonged STAT3 inhibition. In patients who are not predicted to benefit from STAT3 inhibitor monotherapy, combination with chemotherapeutic and targeted agents is another strategy to overcome resistance to chemotherapeutic agents and improve clinical efficacy compared to monotherapy. 

## 6. Conclusions

Targeting STAT3 continues to be of significant interest, with promising opportunities and substantial challenges. Therapeutic strategies require a nuanced approach that accounts for the multifaceted physiologic role of STAT3, in addition to its potential tumor-specific dual function as an oncologic driver and tumor suppressor. Furthermore, given the potential for STAT3 inhibition to overcome treatment failure, combining STAT3 inhibitors with standard-of-care treatment to prevent the emergence of resistance is a critical aspect of developing sustainable therapies. 

In spite of these challenges, continuous technological advances in drug development and improved understanding of STAT3 biology in preclinical studies and clinical trials will promote translation of scientific insights into clinical benefits for the patient. Ultimately, further studies are needed to optimize STAT3 inhibitor activity and customize therapy based on tumor molecular profile in order to substantially expand the treatment options available to cancer patients.

## Figures and Tables

**Table 1:** STAT3 function in human tumors and cell lines.

Cancer Type	STAT3 Function	References
Multiple myeloma		
Cell lines	Oncogene	Catlett-Falcone R et al., 1999 [12]
Astrocytoma		
Cell lines	Oncogene	Konnikova L et al., 2003 [13]
Breast cancer		
Cell lines	Oncogene	Gritsko T et al., 2006 [60]
Cell lines	Oncogene	Garcia R et al., 1997 [61]
Tumor	Oncogene	Watson CJ et al., 1995 [62]
Tumor	Oncogene	Lo H et al., 2005 [63]
Head and neck squamous cell cancer		
Cell lines	Oncogene	Grandis JR et al., 1998 [54]
Tumor	Oncogene	Pectasides E et al., 2010 [64]
Tumor	Tumor suppressor	Shinagawa K et al., 2017 [65]
Non-small cell Lung Cancer		
Cell lines	Onocogene	Alvarez JV et al., 2006 [56]
Tumor	Tumor suppressor	Grabner et al., 2015 [59]
Glioblastoma		
Cell lines	Tumor suppressor	de la Iglesia N et al., 2009 [66]
Papillary thyroid carcinoma		
Cell lines	Tumor suppressor	Couto JP et al., 2012 [67]
Prostate		
Patient derived xenografts	Tumor suppressor	Pencik J et al., 2015 [68]
Pancreas		
Cell lines	Oncogene	Corcoran RB et al., 2011 [69]
Colorectal		
Cell lines	Tumor suppressor	Lee J et al., 2012 [70]
Acute Myelogenous Leukemia		
Tumor	Oncogene	Benekli M et al., 2002 [71]
Chronic Myelogenous Leukemia		
Cell lines	Oncogene	Mencalha AL., 2012 [72]
Acute Lymphocytic Leukemia		
Tumor	Oncogene	Adamaki M et al., 2015 [73]
B-cell lymphoma		
Cell lines	Oncogene	Turton KB et al., 2015 [74]

**Table 2:** STAT3 inhibitors in on-going clinical trials.

Agent	Trial Number	Target	Type	Tumor type	Phase
**Direct inhibitors**					
TTI-101	NCT03195699	SH2 domain	Small molecules	Advanced Solid Tumors	I
Pyrimethamine	NCT01066663	SH2 domain	Small molecule	CLL, Small Lymphocytic Lymphoma	I/II
Silibinin	NCT05689619	SH2 domain	Small molecule	NSCLC and BC Patients with Single Brain Metastasis	II
**Indirect inhibitors**					
AZD4205	NCT04105010	JAK 1	Small molecule	Peripheral T cell lymphoma (PTCL)	I/II
Itacitinib	NCT03670069	JAK 1	Small molecule	Sarcoma	I
Itacitinib	NCT04358185	JAK1	Small molecule	Advanced hepatocellular carcinoma	I
Ruxolitinib	NCT05592015	JAK1/2	Small molecule	T-cell large granular lymphocyte leukemia	II
WP1066	NCT01904123, NCT04334863	JAK2	Small molecule	Recurrent malignant Glioma or progressive metastatic melanoma in the brain	I
Pacritinib	NCT03645824	JAK2	Small molecule	Myelofibrosis	II
**Combination strategies**					
TTI-101 + palbociclib and aromatase inhibitor	NCT05384119	SH2 domain	Small molecules	Metastatic Hormone Receptor (HR)-Positive and Human Epidermal Receptor 2 (HER2)-Negative Breast Cancer	I/II
AZD9150 + Acalabrutinib	NCT03527147	STAT3 mRNA	Antisense oligonucleotides	Relapsed/refractory aggressive Non-Hodgkin’s Lymphoma (NHL)	I
Fedratinib + Ivosidenib/Enasidenib	NCT04955938	JAK2	Small molecule	Blood cancers that show Isocitrate dehydrogenase (IDH)	I
Ruxolitinib + Decitabine	NCT04282187	JAK1/2	small molecule	Accelerated/blast phase myeloproliferative neoplasms	II
Ruxolitinib and Venetoclax	NCT03874052	JAK1/2	Small molecule	Relapsed or Refractory Acute Myeloid Leukemia	I
Ruxolitinib, Carfilzomib, and Low Dose Dexamethasone	NCT03773107	JAK1/2	Small molecule	Carfilzomib-Refractory Multiple Myeloma	I/II
Ruxolitinib and Pelabresib (CPI-0610)	NCT04603495	JAK1/2	Small molecule	Myelofibrosis	III
Ruxolitinib and CPX-351	NCT03878199	JAK1/2	Small molecule	Accelerated phase or blast phase myeloproliferative neoplasm	I/II

## Abbreviations

STAT3α	Signal Transducer and Activator of Transcription 3α
STAT3β	Signal Transducer and Activator of Transcription 3β
STAT1	Signal Transducer and Activator of Transcription 1
STAT2	Signal Transducer and Activator of Transcription 2
STAT4	Signal Transducer and Activator of Transcription 4
STAT5a	Signal Transducer and Activator of Transcription 5a
STAT5b	Signal Transducer and Activator of Transcription 5b
STAT6	Signal Transducer and Activator of Transcription 6
NTD	N-terminal domain
CCD	Coiled-coil domain
DBD	DNA-binding domain
SH2	SRC-homology-2 domain
TAD	Carboxyl-terminal transactivation domain
IFN	Interferon
IL	Interleukin
JAK1	Janus kinase 1
JAK2	Janus kinase 2
Gp130	Glycoprotein 130
BCL6	B cell lymphoma protein 6
PI3K	Phosphoinositide 3 kinase pathways
MAPK	Mitogen activated protein kinase pathways
ERK	Extracellular receptor kinase pathways
EGF	Epidermal growth factor
FGF	Fibroblast growth factor
IGF	Insulin-like growth factor
Gp130	Glycoprotein 130
Tyr705	Tyrosine 705
PTPs	Protein tyrosine phosphatases
PIAS	Protein inhibitors of activated STAT
SOCS	Suppressors of cytokine signaling
SHP-1/2	Src homology region 2 domain-containing phosphatase 1 and 2
NSCLC	Non-small cell lung cancer
siRNA	Small interfering RNA
Rb	Retinoblastoma protein
EMT	Epithelial-to-mesenchymal transition
Twist1	Twist-related protein 1
bHLH	Basic helix-loop-helix
E-cadherin	Epithelial cadherin
N-cadherin	Neural cadherin
MMPs	Matrix metalloproteinases
HIF1α	Hypoxia-inducible factor-1α
VEGF	Vascular endothelial growth factor
PD-L1	Programmed death-ligand 1
NPCs	Neural precursor cells
PTC	Papillary thyroid carcinoma
TPO	Thyroid peroxidase
Snail1	Snail Family Transcriptional Repressor 1
GSK	Glycogen synthase kinase
HNSCCs	Head and neck squamous cell cancer cell lines
DLTs	Dose limiting toxicities
FDA	U.S. Food and Drug Administration
TUNEL	Transferase-mediated dUTP nick-end labeling
AML	Acute Myelogenous Leukemia
GVHD	Graft versus host disease
ORR	Objective response rate
OSCC	Oral squamous cell carcinoma
RT-PCR	Reverse transcription-polymerase chain reaction
ELISA	Enzyme-linked immunosorbent assay
TKIs	Tyrosine kinase inhibitors
HCC	Human hepatocellular carcinoma
Hep*Pten*^−^	Hepatocyte-specific deletion of Pten
cPR	Confirmed partial response
RECIST	Response Evaluation Criteria in Solid Tumors
CLL	Chronic Lymphocytic Lymphoma
BBI608	Napabucasin
SH2	Src homology region 2
Shp1	Domain-containing phosphatase 1
TKI258	Dovitinib
FGFR1	Fibroblast growth factor receptor 1
FGFR2	Fibroblast growth factor receptor 2
FGFR3	Fibroblast growth factor receptor 3
HBCx2	Xenograft model
siRNAs	Small interfering RNAs
shRNA	Short hairpin RNA
RNAi	RNA interference
dsRNA	Double stranded RNA
Gint4.T-STAT3	Glioblastoma a novel aptamer-siRNA chimera
BTK	Bruton tyrosine kinase
3′-UTR	3′-untranslated region
NHL	Non-Hodgkin’s Lymphoma
IDH	Isocitrate dehydrogenase enzyme
IDH1	Isocitrate dehydrogenase enzyme 1
IDH2	Isocitrate dehydrogenase enzyme 2
